# Effects of high-intensity and moderate-intensity exercise training on cardiopulmonary function in patients with coronary artery disease: A meta-analysis

**DOI:** 10.3389/fcvm.2022.961414

**Published:** 2022-09-20

**Authors:** Liying Zheng, Deng Pan, Yimeng Gu, Rumeng Wang, Yanyan Wu, Mei Xue

**Affiliations:** ^1^National Clinical Research Center for Chinese Medicine Cardiology, Xiyuan Hospital, China Academy of Chinese Medical Sciences, Beijing, China; ^2^Graduate School of Beijing University of Traditional Chinese Medicine, Beijing, China

**Keywords:** coronary artery disease, exercise intensity, peak oxygen uptake, anaerobic threshold, meta-analysis

## Abstract

**Purpose:**

The study aims to evaluate the effects of high-intensity and moderate-intensity exercise training on cardiopulmonary function and exercise endurance in patients with coronary artery diseases (CAD).

**Methods:**

We performed a systematic search of the English and Chinese databases from their inception to March 2022. Randomized controlled trials (RCTs) were included to compare high-intensity and moderate-intensity exercise training on cardiopulmonary function in patients with CAD. The primary outcomes included peak oxygen uptake (peak VO_2_) and anaerobic threshold (AT). The secondary outcomes included left ventricular ejection fraction (LVEF), exercises duration (ED), respiratory exchange ratio (RER), resting heart rate (RHR), peak heart rate (PHR) and oxygen pulse (O_2_ pulse). The continuous variables were expressed as mean differences (MD) along with their corresponding standard deviations (SD), and the I^2^ test was applied in the assessment of heterogeneity.

**Results:**

After systematically literature search, 19 studies were finally selected for our meta-analysis (*n* = 1,036), with 511 patients in the experimental group (high-intensity exercise) and 525 patients in the control group (moderate-intensity exercise). The results showed that high-intensity exercise significantly increased patients' Peak VO_2_ [MD = 2.67, 95% CI (2.24, 3.09), *P* < 0.00001], LVEF [MD = 3.60, 95% CI (2.17, 5.03), *P* < 0.00001], ED [MD = 37.51, 95% CI (34.02, 41.00), *P* < 0.00001], PHR [MD = 6.86, 95% CI (4.49, 9.24), *P* < 0.00001], and O_2_ pulse [MD = 0.97, 95% CI (0.34, 1.60), *P* = 0.003] compared with moderate-intensity exercise. However, there were no significant differences in AT [MD = 0.49, 95% CI (−0.12, 1.10), *P* = 0.11], RER [MD = 0.00, 95% CI (−0.01, 0.02), *P* = 0.56], and RHR [MD = 1.10, 95% CI (−0.43, 2.63), *P* = 0.16].

**Conclusion:**

Our results show that high-intensity exercise training has more significant positive effects compared with moderate-intensity exercise training in improving peak VO_2_, LVEF, ED, PHR and O_2_ pulse in patients with CAD, while no significant differences were observed in AT, RER and RHR. To sum up, high-intensity exercise training is better than moderate-intensity exercise training in improving cardiopulmonary function and exercise endurance in patients with CAD.

**Systematic review registration:**

PROSPERO (CRD42022328475), https://www.crd.york.ac.uk/PROSPERO/.

## Introduction

Cardiovascular diseases are the leading cause of death among non-communicable diseases worldwide (1), in which CAD is known having the highest occurrence (2). Thus, CAD has been recognized as a major public health issue worldwide, causing a heavy economic burden to the society (3). Cardiac rehabilitation is internationally recognized as a Class 1A recommendation to improve the prognosis and quality of life in patients with cardiovascular diseases, including patients who have undergone percutaneous coronary implantation (PCI) and coronary artery bypass grafting (CABG). Exercise training is the principal component of cardiac rehabilitation (4). Studies have shown that regular exercise can effectively control the risk factors associated with cardiovascular diseases, such as lowering blood pressure (5), controlling blood glucose (6), controlling body fat, and weight loss (7). Furthermore, it can also improve cardiopulmonary function in patients with CAD (8), improve vascular endothelial function (9), improve ventricular filling, and reverse ventricular remodeling (10). Thereby, it can reduce the incidence of cardiovascular diseases, improve the quality of life of patients, and reduce all-cause mortality (11). Exercise training mainly includes endurance exercise, resistance exercise and aerobic exercise (12). Aerobic exercise training is further classified into three types of exercise intensities: low, medium and high. Until now, there is no international consensus on the choice of exercise intensity. The United States and Canada prefer medium or high-intensity sports training, while Australia and Japan support low or moderate-intensity exercise training (13). The 2016 European Guidelines for the Prevention of Cardiovascular Disease recommended moderate-intensity continuous aerobic training with high safety as an exercise method for patients with cardiovascular diseases (14). Recently, an increasing number of studies have shown that high-intensity aerobic training has the advantages of short training time and high efficiency compared with long and boring moderate-intensity aerobic training (15). However, high-intensity exercise may trigger cardiac arrest in individuals with cardiovascular disease, especially in sedentary patients or those who have advanced cardiovascular diseases (16, 17). Therefore, it is pertinent and urgent to systematically evaluate the effects of the duration and intensity of exercise training and evaluate its effects on patients, and thus establish an optimal exercise training intensity prescription that optimizes the synergy between the rewards and safety (18).

The cardiopulmonary function is a powerful indicator of body's ability to exercise, diagnose diseases and efficiently evaluate prognosis. It can reflect the ability of the circulatory, respiratory and muscular systems to supply oxygen during continuous physical activity (19). Research evidence suggests that improvements in cardiorespiratory fitness are strongly associated with a reduction in all-cause mortality and cardiovascular mortality. And this is why the American Heart Association (AHA) has identified cardiorespiratory fitness as an important landmark indicator in the assessment and intervention of cardiovascular risks and cardiovascular mortality (20). Randomized controlled trials and meta-analyses have demonstrated that both high and moderate exercise intensity can improve cardiopulmonary function in patients with cardiovascular diseases, enhance exercise tolerance, and improve their quality of life compared with drug therapy alone. However, there are numerous controversies regarding the efficacy and safety of different exercise intensities. Therefore, this study is aimed toward the evaluation of the effects of different exercise intensities (moderate and high) on cardiopulmonary function in patients with CAD, to optimize the exercise mode, and provide actionable recommendations for the improvement of cardiopulmonary function and prognosis.

## Methods

### Registration

The protocol was registered on the International Prospective Register of Systematic Reviews (PROSPERO registration number: CRD42022328475). And our Meta-analysis was performed based on the Preferred Reporting Items for Systematic Reviews and Meta-Analyses (PRISMA) guidelines.

### Search strategy

The following electronic databases were searched systematically: PubMed, EMBASE, the Cochrane Library, China National Knowledge Infrastructure (CNKI), Sino-Med, Chongqing VIP, and the Wanfang database, from their inception to March 2022. Taking PubMed as an example, the search strategy was (((((coronary heart disease) OR (coronary artery bypass grafting)) OR (PCI)) OR (ischemic heart disease)) OR (coronary artery stenosis)) AND (exercise intensity) AND (randomized controlled trial). All retrieved articles are imported into EndNote (English publication) and NoteExpress (Chinese publication).

### Inclusion criteria

Clinical trials that satisfy the following criteria were included in our study: (1) Population: the target population was patients with CAD (including patients who have undergone PCI and CABG), consistent with 2014 ACC/AHA/AATS/PCNA/SCAI/STS the diagnostic criteria for patients with stable ischemic heart disease (21), and there are no restrictions on gender, age or duration of disease; (2) Intervention and comparisons: studies that compared exercise training at high (intervention measures) and moderate (control measures) intensities (the classification of exercise intensity was based on the American College of Sports Medicine Exercise Intensity Classification for Healthy Adults, as shown in Table 1), the intervention time for each exercise intervention time is more than 30 mins (22), and the intervention period is more than 4 weeks (23); (3) Outcomes: Outcome indicators must include at least one of peak oxygen uptake and anaerobic threshold; (4) Study Design: Randomized controlled trials (RCTs).

**Table 1:** American college of sports medicine exercise intensity ratings for healthy adults.

**Strength**	**Relative exercise intensity**					**Absolute exercise intensity**				
	**Oxygen uptake reserve (%)**	**Maximum oxygen uptake (%)**	**Heart rate reserve (%)**	**Maximum heart rate (%)**	**Degree of subjective force (points)**	**Young**^a^ **(<MET)**	**Middle age**^b^ **(MET)**	**Aging**^c^ **(MET)**	**Senior citizens**^d^ **(MET)**	**Static resistance training maximum load (%)**
Very light	<20	<25	<20	<35	<10	<2.4	<2.0	<1.6	<1.0	<30
Light	20–39	25–44	20–39	35–54	10–11	2.4–4.7	2.0–3.9	1.6–3.1	1.1–1.9	30–49
Moderate	40–59	45–59	40–59	55–69	12–13	4.8–7.1	4.0–5.9	3.2–4.7	2.0–2.9	50–69
Heavy	60–84	60–84	60–84	70–89	14–16	7.2–10.1	6.0–8.4	4.8–6.7	3.0–4.24	70–84
Very heavy	≥85	≥85	≥85	≥90	17–19	≥10.2	≥8.5	≥6.8	≥4.25	≥85
Maximum	100	100	100	100	20	12.0	10.0	8.0	5.00	100

Oxygen uptake reserve: maximal oxygen uptake - oxygen uptake when quiet; Maximal oxygen uptake: the absorption and utilization of oxygen when each system of human body develops maximum function in the process of movement can be obtained by the cardiopulmonary endurance test of body fitness detection; Heart rate reserve = maximum heart rate - heart rate at rest; Maximum heart rate = 220 – age; Subjective force: Borg rating scale was used; MET, metabolic equivalent, expressed as oxygen metabolism per minute, 1 MET = 3.5 m·kg^−1^·min^−1^.

a 20–39 years old.

b 40–64 years old.

c 65–79 years old.

d 80 years of age or older.

### Exclusion criteria

(1) Studies involving patients with other serious comorbidities; (2) Repeated studies (when there is an overlapping of patient populations overlapped in multiple studies, only the studies with the largest sample sizes were included).

### Data extraction

The literature screening and data extraction were independently performed by two investigators (Zheng LY and Gu YM) independently. The basic information extracted includes: (1) first author, publication time and country; (2) study characteristics: including the patients' ages, sample size, type of exercise, exercise duration and frequency; (3) intervention and control measures; (4) outcome indicators; (5) the Jadad scale quality scoring.

If there are any discrepancies, a third author is consulted to resolve these differences.

### Risk of bias assessment

The quality of the included studies was independently assessed by two researchers independently and scored using the Jadad scale for quality assessment of randomized controlled trials. The total score on the Jadad scale is 5. And a score of 1–2 indicates low literature quality, while a score of 3–5 indicates high literature quality. The risk of bias for each study was assessed using the risk of bias assessment tool recommended in the Cochrane Systematic Evaluator's Manual 5.3. The evaluation indexes include seven aspects such as: the randomization method, assignment hiding, subject blindness, outcome evaluation blindness, data integrity, selective reporting and other bias. Each item was deemed to either be low risk, unknown risk and/or high risk.

### Data synthesis

Data were synthesized using Review Manager Software (Version 5.3; Nordic Cochrane Center, Cochrane Collaboration). Due to the continuous nature of the extracted data, the analysis comprises mean difference (MD) and standard deviation (SD). X^2^ test and I^2^ statistical test were used to analyze heterogeneity. The value for high heterogeneity was set as I^2^ > 50% and *P* < 0.10, and a random effects model was used to evaluate the combined effect size of included data (24); however, if I^2^ < 50% and *P* > 0.10, heterogeneity was considered small and the fixed effects model was used. In case of large heterogeneity, the sources of heterogeneity were examined through sensitivity and subgroup analyses. Publication bias was assessed using funnel plots.

## Results

### Study selection

**Figure 1:**
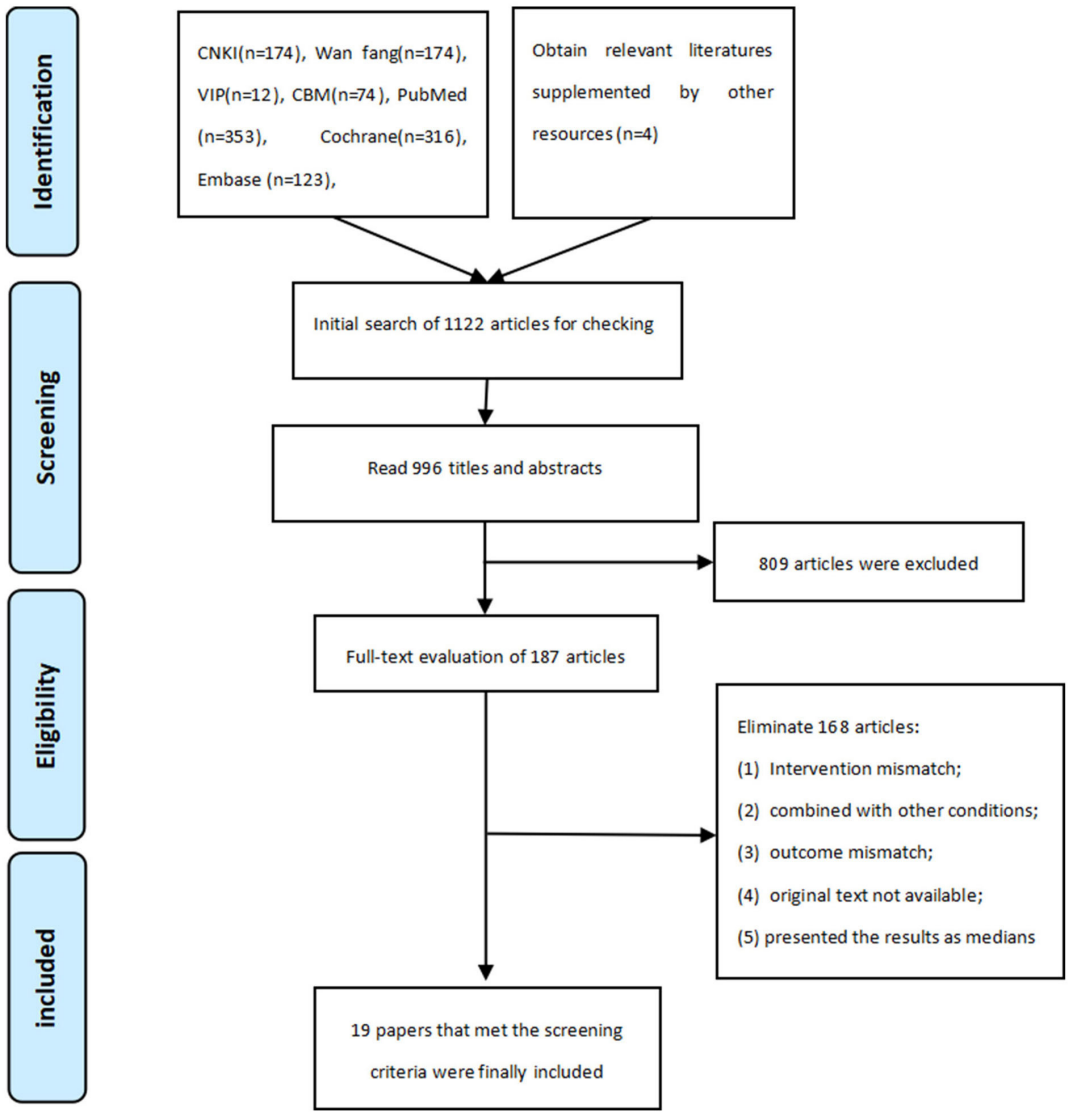
Flow diagram of literature search and selection process.

Our literature search is started from the inception of the aforementioned databases to March 2022. The literature screening flow chart of literature screening is shown in Figure 1. A total of 1,225 literatures were retrieved, which comprises 434 Chinese literature and 791 English literature, and four literatures was supplemented by manual retrieval from other sources. We imported the literature into EndNote X9 and NoteExpress (both are literature management software) and eliminated 233 duplicate literature. Furthermore, 809 articles were excluded by reading the titles and abstracts, subsequently, we perused through the remaining 187 articles, and finally selected 19 articles that met the inclusion criteria (25–43).

### Study characteristics

Nineteen trials were included (Table 2) involving a total of 1,036 patients which include 511 in the high-intensity exercise training group and 525 in the moderate-intensity exercise training group. 7 literatures (31, 32, 36, 38, 39, 42, 43) had an intervention period of <12 weeks, and 12 literatures (25–30, 33–35, 37, 40, 41) had an intervention period of 12 weeks or more.

**Table 2:** Characteristics of included studies.

**References**	**Publish time**	**Country**	**Sample size**		**Gender**	**Age**		**Type of movement**	**Exercise time**	**Exercise prescription**		**Closing indicators**	**Quality score**
			**Test group**	**Control group**	**(Male/** **Female)**	**Test group**	**Control group**			**Test group**	**Control group**		
Luan et al. (25)	2018	China	41	41	T:34/7 C:32/9	59.73 ± 7.9	59.67 ± 7.93	Power bikes	12 weeks, 3 times/week	(1) The initial exercise load was 60% of the peak power in cardiopulmonary exercise test, and the adaptive training lasted for 1 week, 3 times per week; (2) 80% of the peak power was used for treadmill exercise, which was carried out in interval training mode (3-min training, 1-min rest), 10 sets per time, a total of 40 mins; 3 times per week	The exercise load was 60% of the peak power in cardiopulmonary exercise test, 40 min/time, 3 times per week	1, 2, 3, 4	0
Ju et al. (26)	2018	China	25	25	–	56.64 ± 9.86	56.64 ± 9.86	Power bikes	12 weeks, 3 times/week	80% of the peak power was used for treadmill exercise, which was carried out in interval training mode (3-minute training, 1-minute rest), 10 sets per time, a total of 40 mins; 3 times per week	60% of PP for exercise load power treadmill exercise, intermittent training mode (3-min training, 1-min rest), 10 groups/time, a total of 40 mins; 3 times per week	1, 2, 3, 4	2
Gao et al. (27)	2015	China	22	21	T:16/5 C:18/4	59.4 ± 7.9	61.2 ± 8.0	Power bikes	12 weeks, 3 times/week	80% of PP for exercise load power treadmill exercise, intermittent training mode (3-min training, 1-min rest), 10 groups/time, a total of 40 mins; 3 times per week	60% of PP for exercise load power treadmill exercise, intermittent training mode (3-min training, 1-min rest), 10 groups/time, a total of 40 mins; 3 times per week	1, 2, 3, 4	2
Wang et al. (28)	2010	China	22	27	T:15/7 C:18/9	65.5 ± 6.9	68.7 ± 7.0	Tai ji /Jogging	24 weeks	Jogging, exercise intensity ≥ 70% VO_2max_, available for 4 weeks to gradually reach that exercise intensity	Tai ji practice 40 min per day, 5 d per week, exercise time about 200 min per week	1, 4, 7, 8	0
Zhang et al. (29)	2022	China	22	21	T:16/6 C:13/8	58.1 ± 13.61	62.10 ± 10.24	Power bikes	12 weeks, 3 times a week	80% of the peak power was used for treadmill exercise, which was carried out in interval training mode (3-minute training, 2-min rest), 40 mins in total; 3 times per week	60% of PP for exercise load power treadmill exercise, intermittent training mode a total of 40 mins; 3 times per week	1, 2, 3	2
Gu et al. (30)	2020	China	23	26	T:15/8 C:17/9	64.1 ± 9.2	66.5 ± 7.8	Power bikes	12 weeks, 5 times a week	(1) 70% of the peak power (PP) was used as exercise load for power treadmill training, starting with 0 W power, warming up for 3 min without power, and then increasing with a certain load range, so that patients could reach the target power within 8–10 min. During the whole process, the patient maintained a rotational speed of 55–65 r/min for treadmill exercise until reaching 70% of the maximum power assessed by the patient; (2) 70% of the maximum power is used as a constant power treadmill until the 25th minute, and the last 5 mins of recovery time. A total of 30 mins, 5 times a week	(1) 50% of the peak power is used as the exercise load for power treadmill training, resting for 5 min, starting at 0 W, warming up for 3 min without power, and then with a certain amount (specific power varies from person to person, ensuring 8–10 min to reach the target power), patients performed treadmill exercise at a rotational speed of 55–65 r/min until 50% of the maximum power assessed by the patient power as the treadmill of constant power until the 25th minute, the last 5 mins to resume, a total of 30 mins, 5 times a week training was achieved; (2) Take 50% of the maximum	1, 2, 3, 6, 7	2
Rognmo et al. (31)	2004	Norway	8	9	T:6/2 C:8/1	62.9 ± 11.2	61.2 ± 7.3	Treadmill	10 weeks, 3 times a week	A total of 33 min: (1) 5-min warm-up period at an intensity corresponding to 50–60% of VO_2_peak (65–75% of HRpeak); (2) walking four intervals of 4 min at 80–90% of VO_2_peak (85–95% of HRpeak), the intervals 3 min of walking at 50–60% of VO_2_peak	41 mins continuous exercise at an intensity of 50–60% of VO_2peak_, representing the same total training load as the high intensity aerobic exercise group	1, 5, 6, 7	5
Moholdt et al. (32)	2009	Norway	28	31	T:24/4 C:24/7	60.2 ± 6.9	62.0 ± 7.6	Treadmill	4 weeks, 5 times/week	Total time 38 mins: (1) 8 mins warm-up; (2) 4 times of 4-min intervals with HR at 90% of maximum HR, with active pauses of 3 mins of walking at the exercise session was terminated by 5 mins cool-down	Walked continuously at 70% of maximum HR for 46 mins to ensure isoenergetic training protocols	1, 3, 5, 6	4
Benetti et al. (33)	2010	Brazil	29	29	-	57.7 ± 6.1	57.7 ± 6.1	Treadmill	12 weeks, 5 times a week	Total time 45 mins: (1) patients exercised at around 85% of their maximum heart rate (HR) achieved in the stress test.	Total time 45 mins: patients exercised at a ~75% of their HRmax.	1	2
Conraads et al. (34)	2015	Belgium	100	100	T:91/9 C:89/11	57.0 ± 8.8	59.9 ± 9.2	Treadmill and bicycle	12 weeks, 3 times per week	Total time 38 min: (1) 60–70% peak HR for 10 min warm-up, followed by 4 × 4 min training at 85–95% peak HR; (2) 50–70% peak active interval training was performed at an interval of 4 × 3 min performed at an interval of 4 × 3 min	Total time: 5 min warm-up at 60–70% Peak HR, followed by 37 min continuous training at 70–75% Peak HR and 5 min relaxation at 60–70% peakHR	1, 5, 6, 7, 8	2
Katharine et al. (35)	2014	United States	15	13	–	60 ± 7	58 ± 9	Treadmill	24 weeks	(1) 5-min period of active warm-up; (2) 3-min period of training at 60–70% of heart rate reserve, (3)4 higher-intensity work A 3-min recovery period set at an intensity of 60–70% of heart rate reserve followed each of the 4 higher-intensity work intervals of 4 mins each, set at an intensity corresponding to 80–90% of heart rate reserve. A 3-minute recovery period set at an intensity of 60–70% of heart rate reserve followed each of the four higher-intensity intervals.	A 5-min period of active warm-up, 30 mins of cardiorespiratory training, and 5 mins of active cool-down. The exercise intensity was prescribed at 60–80% of heart rate reserve throughout	1, 6, 7	2
Koldobika et al. (36)	2016	Spain	36	36	–	58 ± 11	58 ± 11	Power bikes	8 weeks	Total time: 40 min: (1) 5–12 min warm up (25% PeakWR); (2) (15–30 groups) × 20 s interval (120–125% PeakWR); (3) (15–30 groups) × 40 s rest (25% PeakWR); (4) 5–13 min relaxation (25% PeakWR)	Total time of use: 40 min: (1) 5–12 min heat body; (2) 15–30 min continuous training (VT1~VT1+10%);Relax for 5–13 mins	1, 4, 5, 6, 7	4
Lee et al. (37)	2019	Canada	17	14	Female	69.3 ± 9.9	69.6 ± 5.9	Walking/ Jogging	24 weeks, 5 times a week	(1) A warm-up period of 5–10 min of walking performed at 60–70% of Peak HR, and/or RPE of 10–12 on the 6–20 Borg Scale; (2) four 4-min intervals of walking/jogging performed at an intensity targeting 90–95% of Peak HR, and/or RPE ≥17 on the Borg Scale, interspersed with 3 min of active recovery performed at an intensity of 50–70% of Peak HR; (3) a cool-down period of 5 min of walking performed at an intensity of 50–70% of Peak HR, and/or RPE ~10–12 on the Borg Scale.	Jog for about 30–40 mins at 60–80% of peak oxygen uptake, with a warm up and rest period	1, 5, 6, 7	5
Koldobika et al. (38)	2019	Spain	57	53	T:50/7 C:42/11	57.6 ± 9.8	58.3 ± 9.5	Power Bikes	8 weeks, 3 times a week	Total time: 40 min: (1) 5–12 min warm up (25% PeakWR); (2) (15–30 groups) × 20 s interval (120–125% PeakWR); (3) (15–30 groups) × 40 s rest (25% PeakWR); (4) 5–13 min relaxation (25% PeakWR)	Total time of use: 40 min: (1) 5–12 min heat body; (2) 15–30 min continuous training (VT1~VT1+10%); (3) relax for 5–13 mins	1, 4, 5, 7	4
Keteyian et al. (39)	2014	United States	15	13	T:11/4 C:12/1	60.0 ± 7.0	58.0 ± 9.0	Treadmill	4 weeks, 5 times a week	(1) 5-min period of active warm-up, (2) 3-min period of training at 60–70% of heart rate reserve and then 4 higher-intensity work a 3-min recovery period set at an intensity of 60–70% of heart rate reserve followed each of the 4 higher-intensity work intervals of 4 mins each, set at an intensity corresponding to 80–90% of heart rate reserve. (3) 3-min recovery period set at an intensity of 60–70% of heart rate reserve followed each of the 4 higher intensity intervals.	5-min period of active warm-up, 30 min of cardiorespiratory training, exercise intensity was prescribed at 60–80% of heart rate reserve throughout, and 5 mins of active cool-down	1, 4, 5, 6, 7, 8	5
Currie et al. (40)	2013	Canada	11	10	–	62 ± 11	68 ± 8	Power bikes	12 weeks, 3 times/week	Total time of use: 30 min: (1) 5 min heat body; (2) 10 × 1 min interval (89–102–110% PeakWR); (3) 10 × 1 min rest (10% peakWR); (4) relax in 5 mins	Total use time: 30–50 mins: (1) 5 min heat body; (2) continued training for 30–50 min (58%PeakWR); (3) relax for 5 mins	1, 5, 7	3
Cardozo et al. (41)	2015	Brazil	23	24	–	56 ± 12	62 ± 12	Treadmill	16 weeks, 3 times/week	Total time: 40 min: (1) 5 min warm-up; (2) 8 × 2 min interval (90% HRmax); (3) 7 × 2 min rest (60% HRmax); (4) relax in 5 mins	Total duration: 40 min (1) 5 mins heat body; (2) 30 mins continued training (70–75.0% HRmax); (3) relax for 5 mins	1, 5, 7, 8	3
Kim et al. (42)	2015	Korea	14	14	T:12/2 C:10/4	60.0 ± 13.7	57.0 ± 11.9	Treadmill	6 weeks, 3 per week times	A total of 45 mins: (1) 10-min warm-up at 50–70% of HRR, (2) four times of 4-min intervals of walking on a treadmill at 85–95% of HRR with three active pauses of 3-min walking at 50–70% of HRR, and a 10-min cooldown at 50–70% of HRR	A total of 45 mins: (1) 10-min warm-up, (2) 25-min walk on a treadmill continuously at 70–85 % of HRR and a 10-min cooldown	1, 5, 6, 7	2
Gremeaux et al. (43)	2014	France	9	10	T:7/2 C:7/3	59.2 ± 8.1	59 ± 7.4	Treadmill and bicycle	7 weeks, 3 times a week	(1) 5 mins at 50% of the graded maximal exercise test maximal HR; (2) patients performed one set of 18 mins composed of repeated phases of 3 consecutive 6-min; (3) patients had a 3-min cool down period	(1) Patients performed a 5-min warm-up at 50% of the maximal HR measured; (2) a continuous workout at 70% of the maximal HR measured on the graded maximal exercise test for 18 mins, followed by 3 mins of active recovery	1	0

(1) Peak oxygen uptake (Peak VO_2_); (2) anaerobic threshold (AT); (3) left ventricular ejection fraction (LVEF); (4) exercises duration (ED); (5) respiratory exchange ratio (RER); (6) resting heart rate (RHR); (7) peak heart rate (PHR); (8) oxygen pulse (O_2_ pulse).

### Risk of bias

**Figure 2:**
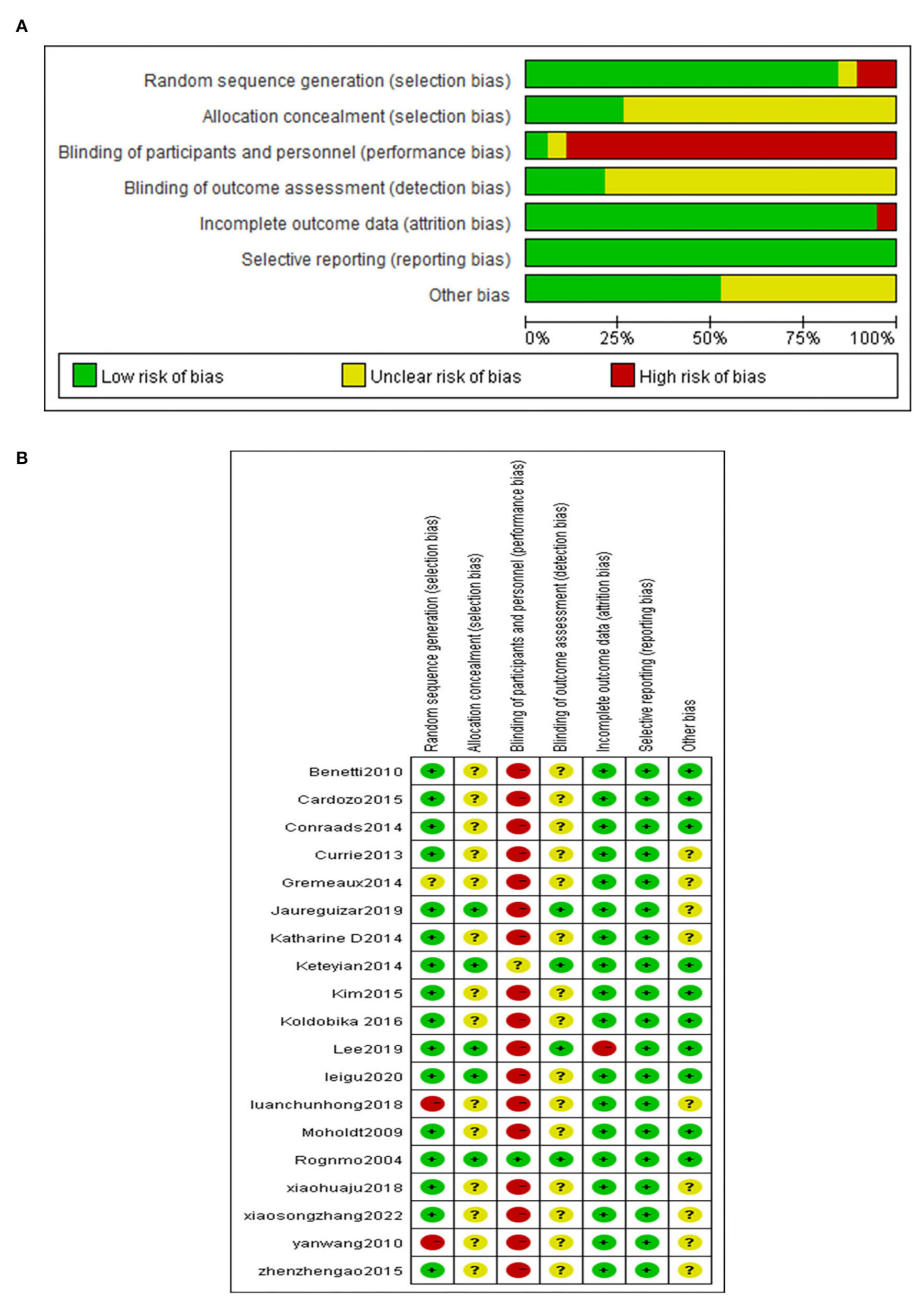
**(A)** Overall analysis diagram. **(B)** Single literature analysis diagram. “+” is low risk. “?” is unclear, and “–” is high risk.

The quality assessment method of the 19 selected literatures is shown in Figure 2. 16 literatures (25–27, 29, 30, 32–42) described specific randomization methods, and 3 literatures (28, 31, 43) adopted a semi-randomization method combined with patients' wishes, and 5 literatures (30, 31, 37–39) implemented the allocation concealment; Considering the risk associated with exercise, patients are required to give and informed consent, so only one literature (31) implemented blind method for patients, five literatures (31, 37–39, 42) adopted the blinded outcome assessors; 13 (31–43) literatures recorded the circumstances and reasons for subjects who were lost to follow-up and dropped out of the trial. There were no selective reporting of outcome indicators, and no significant bias was identified in risk assessment.

### Results of meta-analysis

#### Peak oxygen uptake (peak VO_2_)

**Figure 3:**
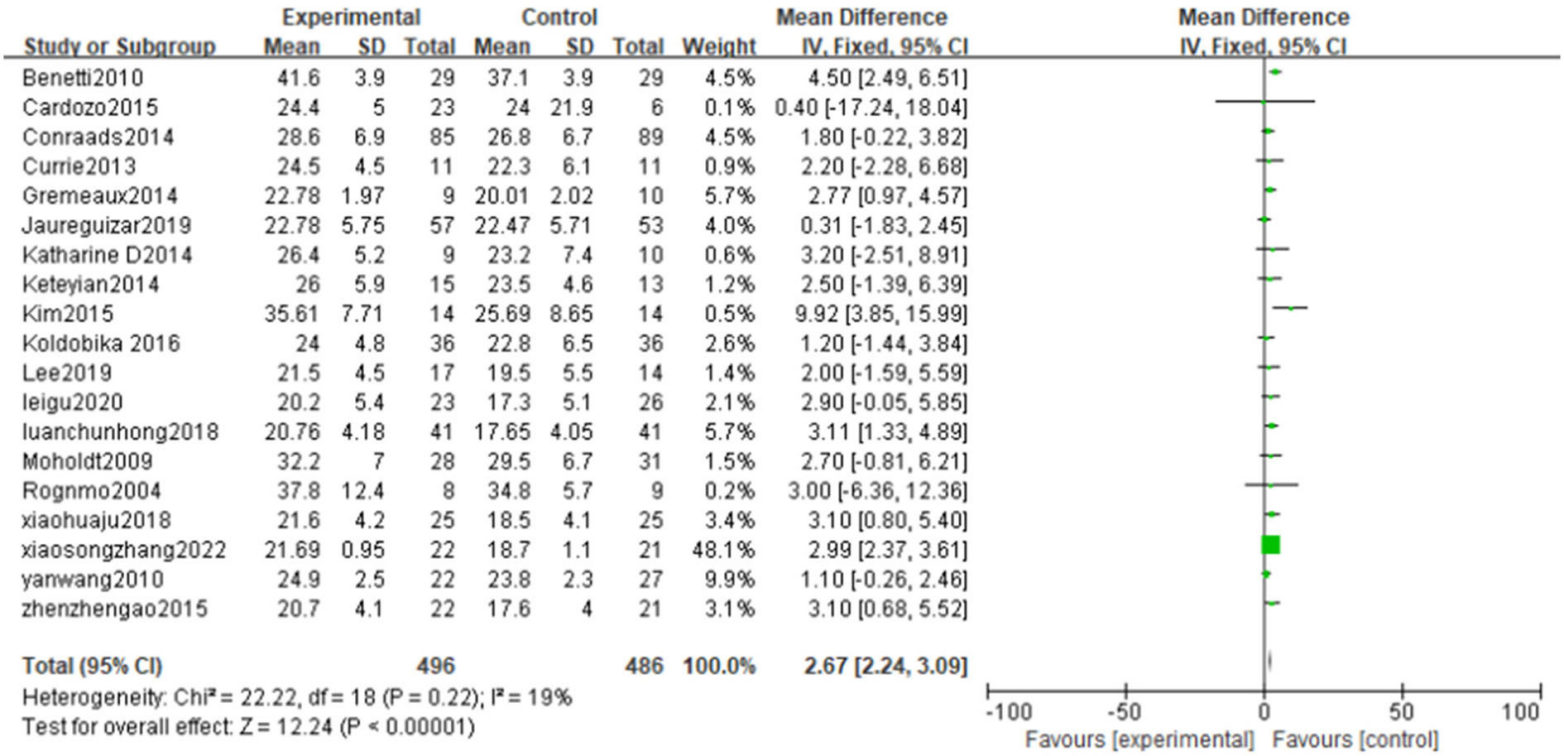
Forest plot comparing the improvement of peak VO_2_ between two exercise intensity.

Peak VO_2_ is reported in all the 19 included literatures (25–43) high-intensity exercise training on Peak VO_2_ showed significantly better effects than that of moderate-intensity exercise training [MD = 2.67, 95% CI (2.24, 3.09), *P* < 0.00001, I^2^ = 19%] (Figure 3).

#### Anaerobic threshold

**Figure 4:**
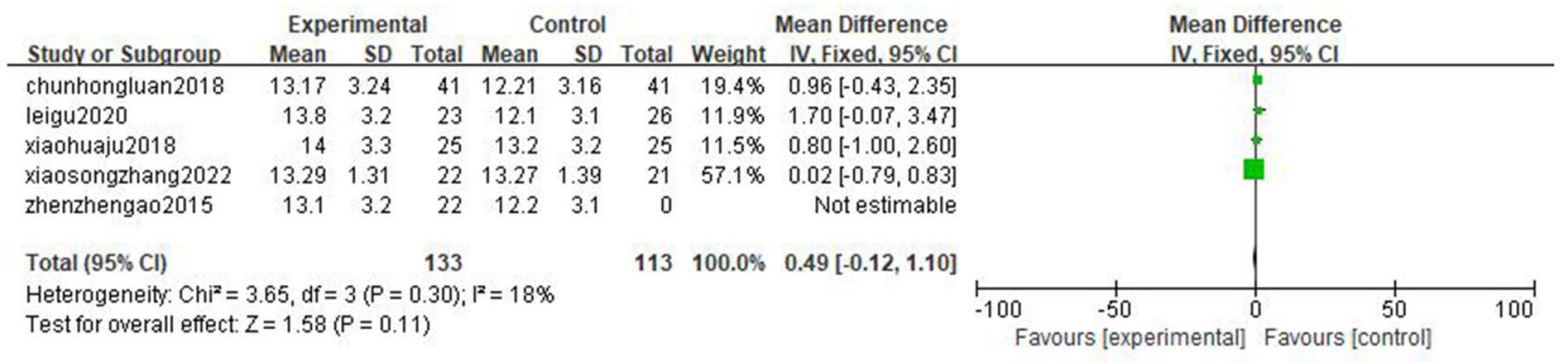
Forest plot comparing the improvement of AT between two exercise modes.

Five literatures (25–27, 29, 30) reported anaerobic threshold (AT), and the results showed that there are no statistically significant difference in the improvement effect of two types of exercise intensity on AT [MD = 0.49, 95%CI (−0.12, 1.10), *P* = 0.11, I^2^ = 18%] (Figure 4).

#### Left ventricular ejection fraction

**Figure 5:**
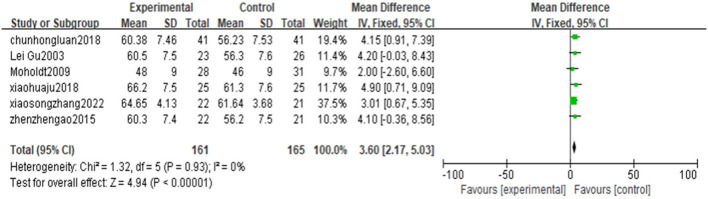
Forest plot of the effects of high-intensity and moderate-intensity exercise on LVEF in CAD patients.

Six articles were reported on left ventricular ejection fraction (LVEF) (25–27, 29, 30, 32), and the result shows that high-intensity exercise training has a significantly better effect on LVEF than moderate-intensity exercise training [MD = 3.60, 95% CI (2.17, 5.03), *P* < 0.00001, I^2^ = 0%] (Figure 5).

#### Exercise duration

**Figure 6:**
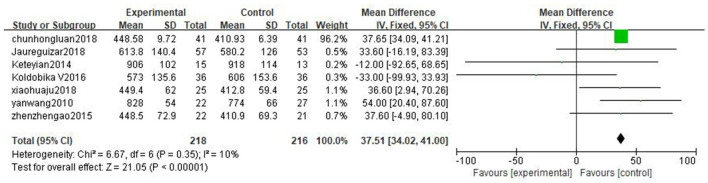
Forest plot comparing the effects of two exercise modes on ED improvement.

Seven literatures (25–28, 36, 38, 39) reported exercise duration (ED). The result shows that high-intensity exercise has significant effects on ED compared to moderate-intensity exercise training [MD = 37.51, 95%CI (34.02, 41.00), *P* < 0.00001, I^2^ = 10%] (Figure 6).

#### Respiratory exchange ratio

**Figure 7:**
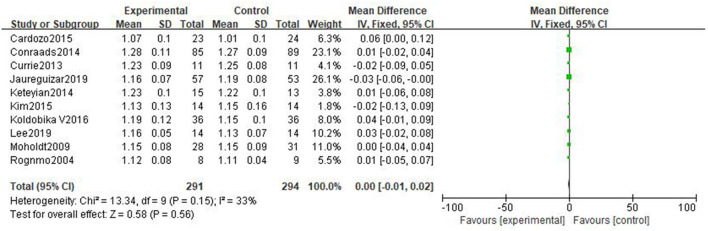
Forest plot comparing the effects of two exercise modes on RER improvement.

Ten studies (31, 32, 34, 36–42) reported respiratory exchange ratio (RER) with little heterogeneity. The result shows that there is no significant difference in the improvement effect of the two exercise intensities on RER [MD = 0.00, 95%CI (−0.01, 0.02), *P* = 0.56, I^2^ = 33%] (Figure 7).

#### Resting heart rate

**Figure 8:**
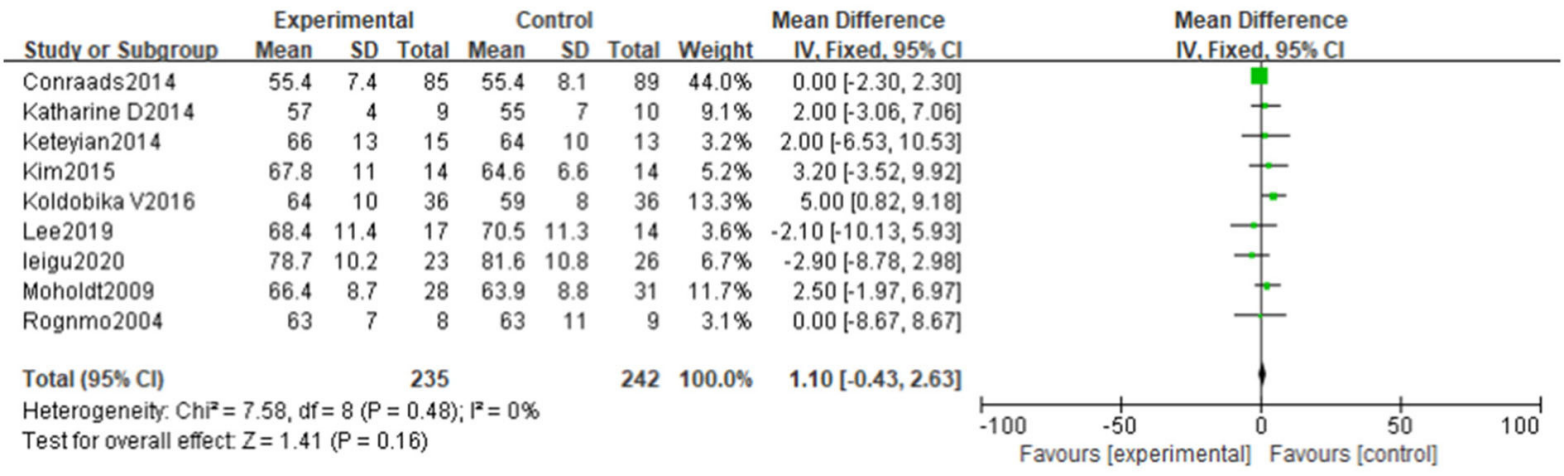
Forest plot of the effects of high-intensity and moderate-intensity exercise on RHR in CAD patients.

Nine literatures (30–32, 34–37, 39, 42) reported resting heart rate (RHR), and the results shows that there is no significant difference in the improvement effect of RHR between the two exercise intensities [MD = 1.10, 95% CI (−0.43, 2.63), *P* = 0.16, I^2^ = 0%] (Figure 8).

#### Peak heart rate

**Figure 9:**
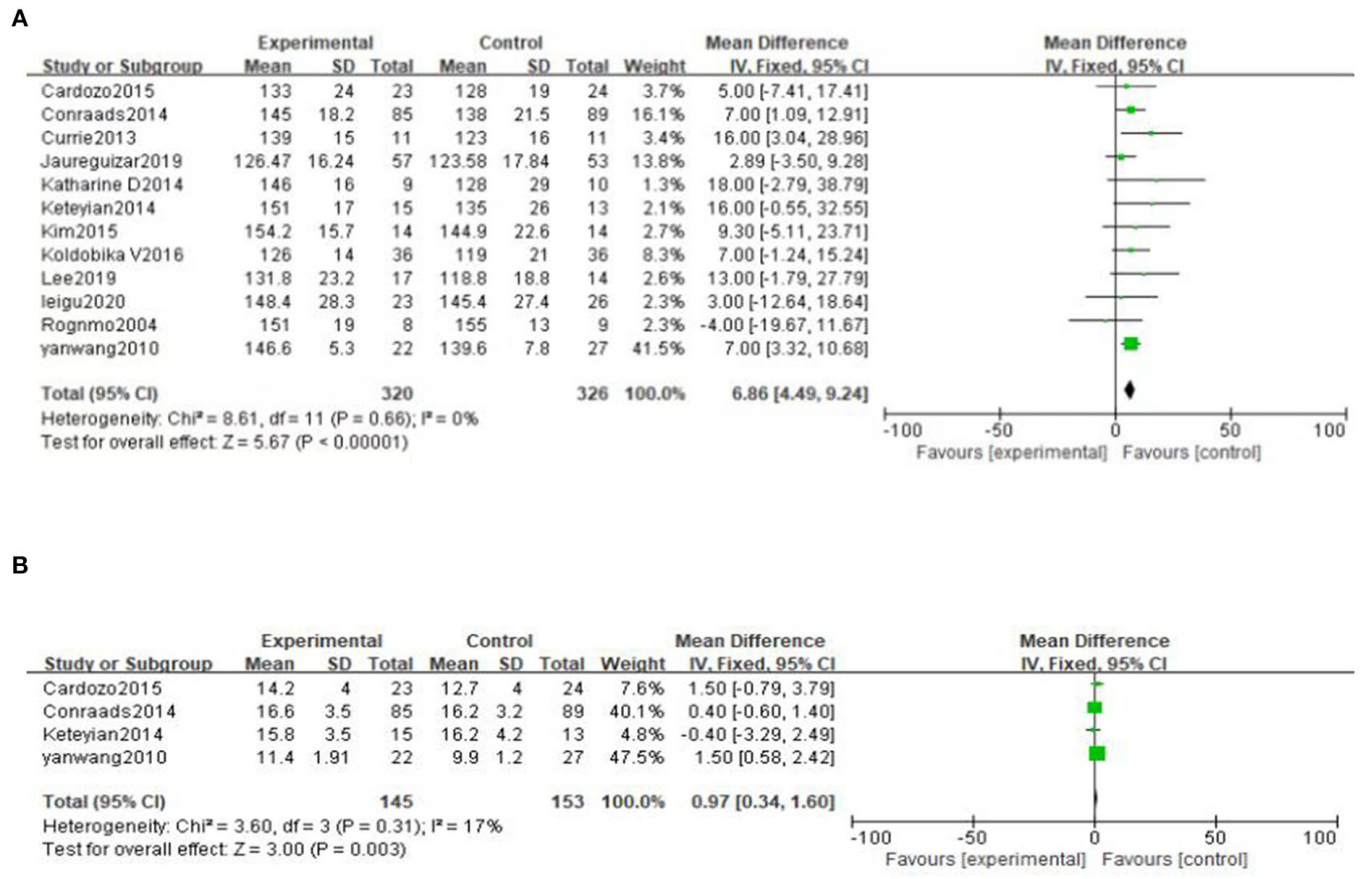
**(A)** Forest plot of the effects of high-intensity and moderate-intensity exercise on PHR in CAD patients. **(B)** Forest plot comparing the improvement effect of two exercise modes on O_2_ pulse.

Twelve studies (28, 30, 31, 34–42) reported peak heart rate (PHR). The result showed that high-intensity exercise training has a significantly effect on PHR compared with moderate-intensity exercise training [MD = 6.86, 95% CI (4.49, 9.24), *P* < 0.00001, I^2^ = 0%] (Figure 9A).

#### Oxygen pulse

O_2_ pulse is reported in 4 literatures (28, 34, 39, 41). The result shows that high-intensity exercise training has a significant improvement on O_2_ pulse compared to moderate-intensity exercise training [MD = 0.97, 95%CI (0.34, 1.60), *P* = 0.003, I^2^ = 17%] (Figure 9B).

#### Subgroup analysis

**Figure 10:**
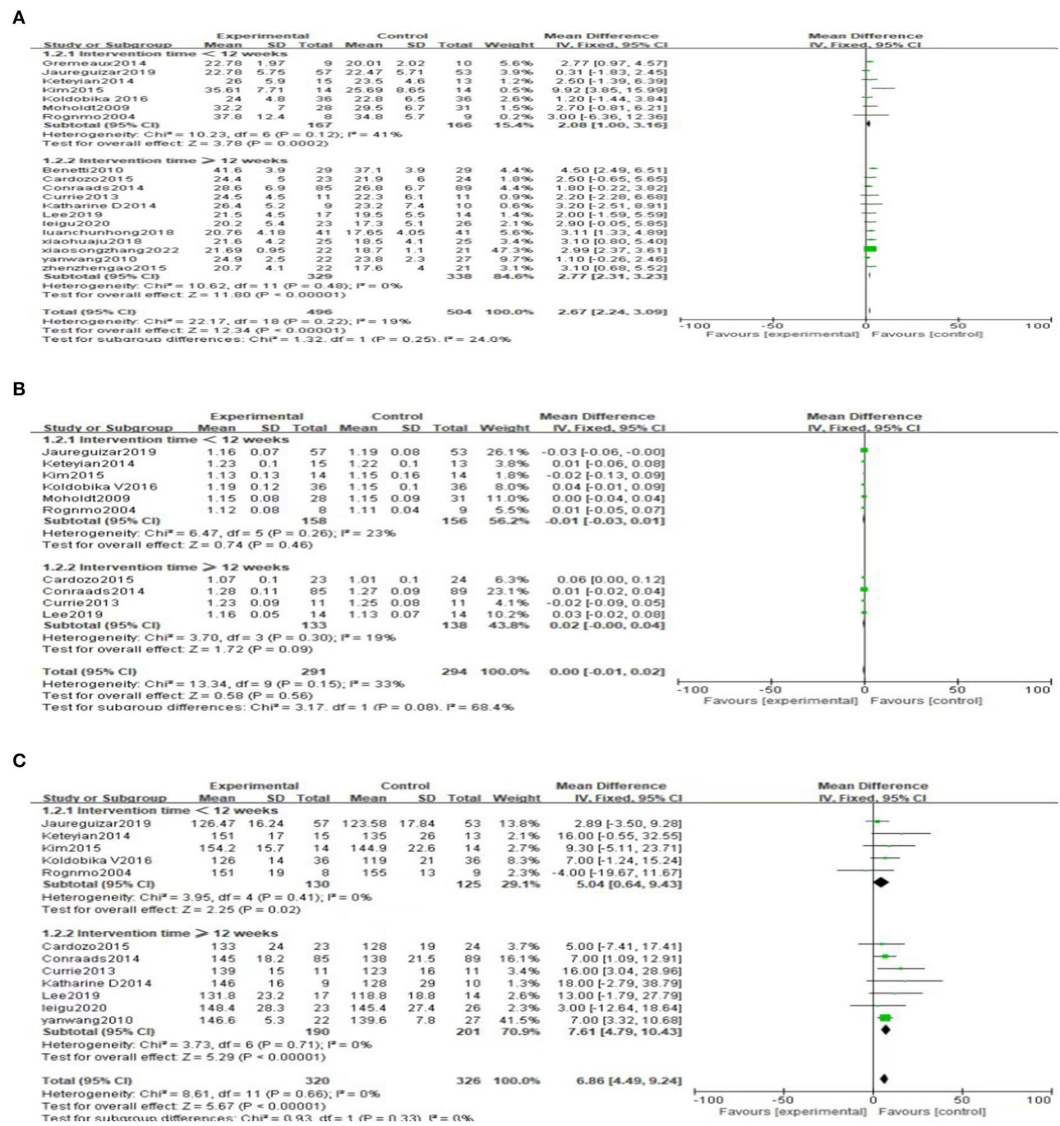
**(A)** Subgroup analysis of peak VO_2_. **(B)** Subgroup analysis of RER. **(C)** Subgroup analysis of PHR.

**Figure 11:**
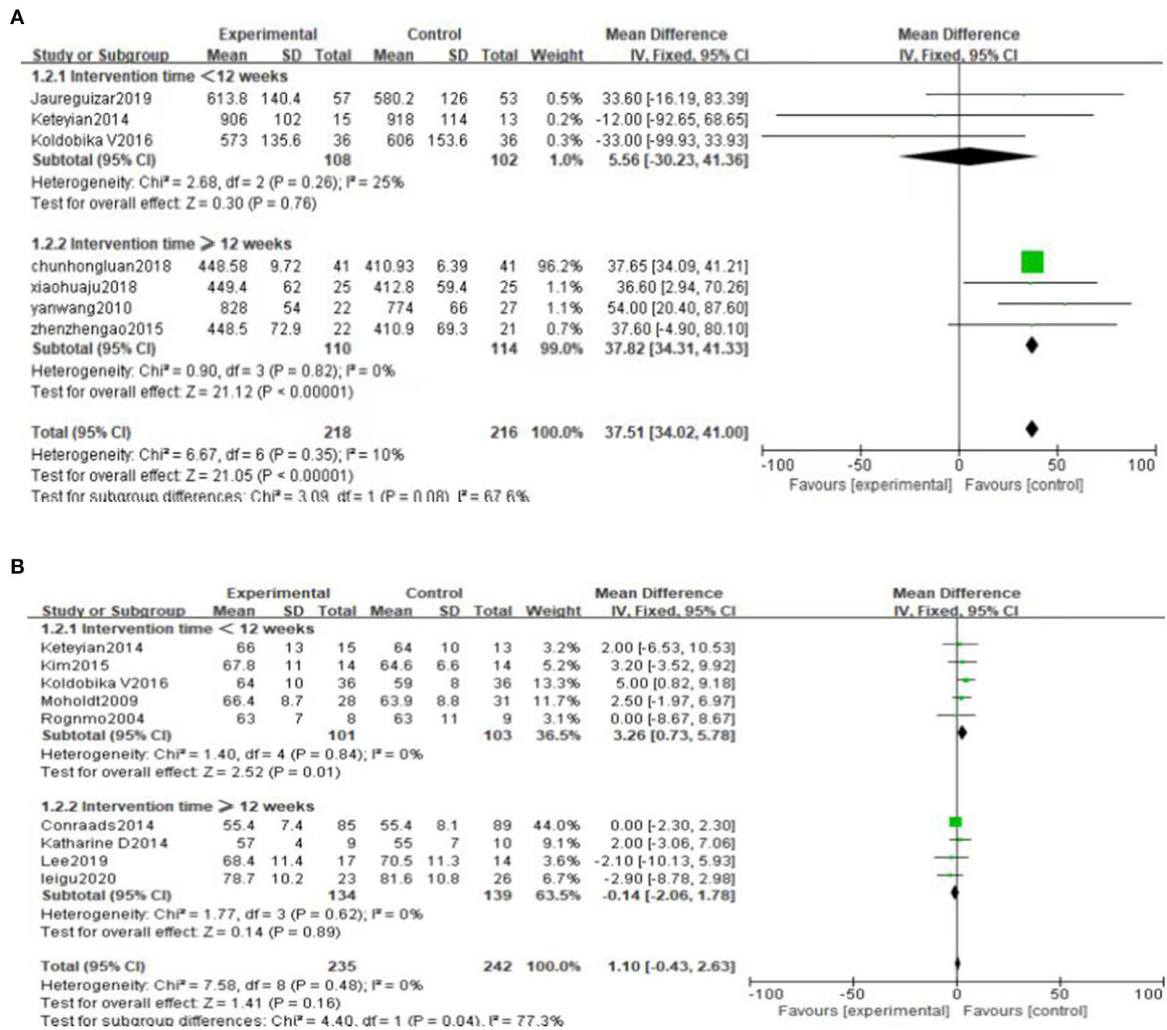
**(A)** Subgroup analysis of ED. **(B)** Subgroup analysis of RHR.

Subgroup analysis was performed based on the duration of the intervention. We set 12 weeks as the boundary (one group was <12 weeks, the other group was ≥12 weeks). The results of subgroup analysis showed no significant difference in the influence of intervention time on Peak VO_2_, RER and PHR, and the results of subgroup analysis were consistent with the results of overall analysis (Figure 10). Interestingly, the subgroup analysis of ED showed no statistically significant difference when intervention duration was <12 weeks [MD = 5.56, 95%CI (−30.23, 41.36), *P* = 0.76]. However, when intervention time was ≥12 weeks, the difference was statistically significant [MD = 37.82, 95%CI (34.31, 41.33), *P* < 0.00001] (Figure 11A). Subgroup analysis of RHR showed that the difference was statistically significant when intervention time was <12 weeks, and high-intensity was better than moderate-intensity [MD = 3.26, 95%CI (0.73, 5.78), *P* = 0.01]. However, there was no significant difference when intervention time was ≥12 weeks [MD = −0.14, 95%CI (−2.06, 1.78), *P* = 0.89] (Figure 11B).

#### Publication bias

**Figure 12:**
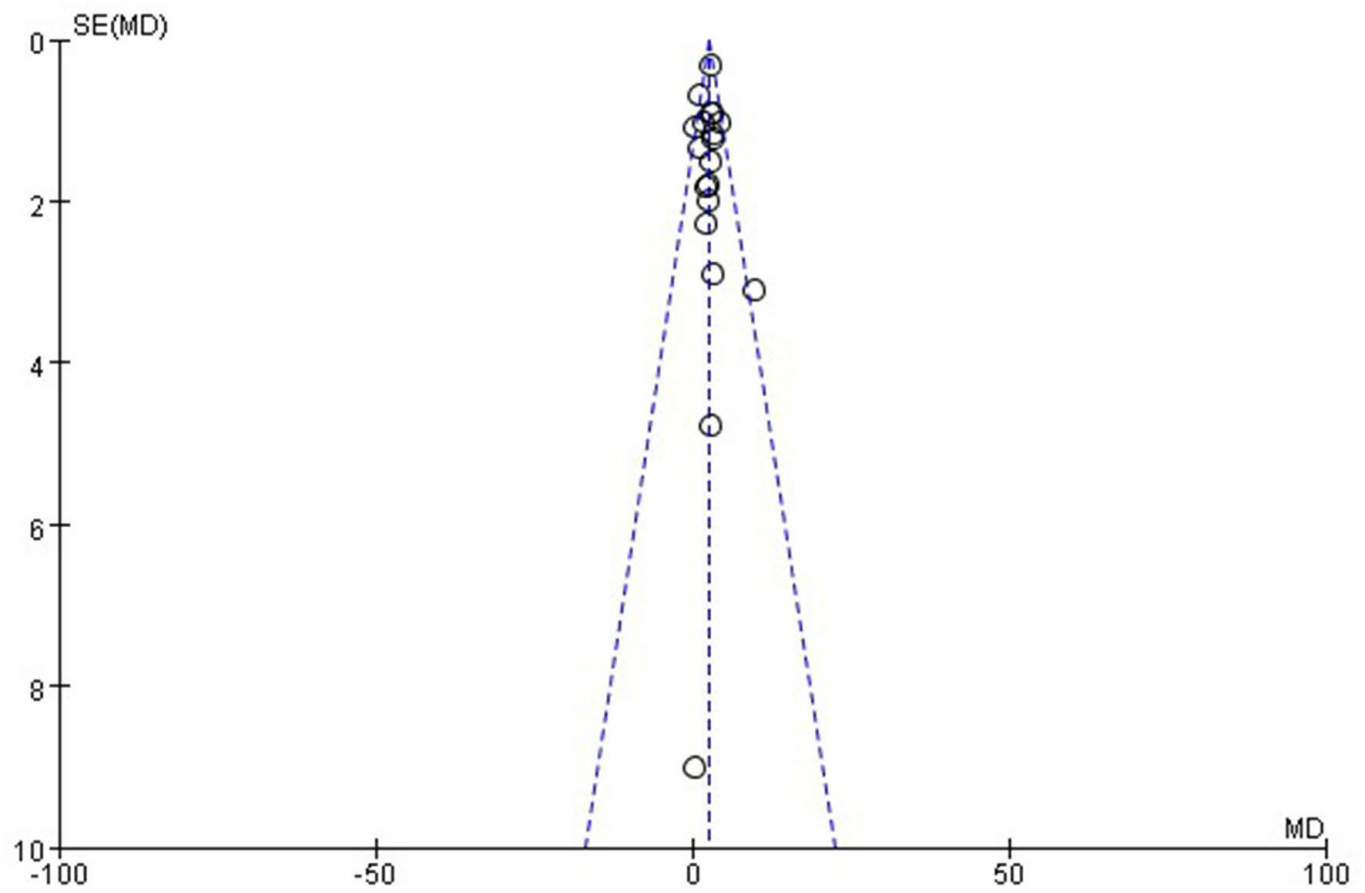
Funnel chart of publication bias.

The funnel plot (Figure 12) was generated to reflect the publication bias. It can be seen from the figure that the distribution of all included studies is relatively concentrated and basically all data are located in the funnel plot. Therefore the possibility of publication bias of this study is small.

### Sensitivity analysis

We performed sensitivity analysis to evaluate the influence of any individual study on the overall effect. For AT, the difference was statistically significant when Zhang et al. (29) was removed, and high-intensity was better than moderate-intensity [MD = 1.12, 95%CI (0.19, 2.06), *P* = 0.02, I^2^ = 0%]. For RHR, the difference was statistically significant when Conraads et al. (34) was removed, and high-intensity group showed slightly better than moderate-intensity group [MD = 2.10, 95%CI (0.13, 4.07), *P* = 0.04, I^2^ = 0%]. However, there is no significant difference in the improvement effect of the two exercise intensities on O_2_ pulse when Wang et al. (28) was removed [MD = 0.49, 95%CI (−0.38, 1.36), *P* = 0.27, I^2^ = 0%].

### GRADE assessment

Table 3 shows the GRADE assessment of the certainty on the effect of exercise intensity in patients with CAD. Peak VO_2_, AT, LVEF, and RER were rated as moderate, while ED, RHR, PHR, and O_2_ pulse were rated as low-quality evidence. Reasons for downgrading: (1) For the risk of bias, only three literatures (28, 31, 43) refer to patients' wishes using a semi-randomized method; most studies do not specify whether allocation concealment is implemented; Considering the risk associated with exercise, patients are required to give and informed consent, so only one literature (31) implemented blind method for patients. (2) In terms of inconsistency: the results of two studies (36, 39) were inconsistent for the effect of exercise intensity on ED. For RHR, two studies (30, 37) had inconsistent results. For PHR, there was one study (31) with different results. For O_2_ pulse, one study (39) showed different results from others. And these may be due to differences in study population, gender, and duration of intervention.

**Table 3:** Grading of Recommendations Assessment, Development, and Evaluation (GRADE) assessment.

**Quality assessment**							**No of patients**		**Effect**		**Quality**	**Importance**
**No. of studies**	**Design**	**Risk of bias**	**Inconsistency**	**Indirectness**	**Imprecision**	**Other considerations**	**High-intensity vs. moderate-intensity**	**Control**	**Relative (95% CI)**	**Absolute**		
**Peak oxygen uptake (better indicated by lower values)**												
19	Randomized trials	Serious^a^	No serious inconsistency	No serious indirectness	No serious imprecision	None^b^	496	486	–	MD 2.67 higher (2.24–3.09 higher)	⊕⊕⊕○	Critical
											Moderate	
**Anaerobic threshold (better indicated by lower values)**												
5	Randomized trials	Serious^a^	No serious inconsistency	No serious indirectness	No serious imprecision	None	133	113	–	MD 0.49 higher (0.12 lower to 1.1 higher)	⊕⊕⊕○	Critical
											Moderate	
**Left ventricular ejection fraction (better indicated by lower values)**												
7	Randomized trials	Serious^a^	No serious inconsistency	No serious indirectness	No serious imprecision	None	169	174	–	MD 3.7 higher (2.28 to 5.11 higher)	⊕⊕⊕○	Important
											Moderate	
**Exercises duration (better indicated by lower values)**												
7	Randomized trials	Serious^a^	Serious^c^	No serious indirectness	No serious imprecision	None	218	216	–	MD 37.51 higher (34.02 to 41 higher)	⊕⊕	Important
											Low	
**RER (better indicated by lower values)**												
10	Randomized trials	Serious^a^	No serious inconsistency	No serious indirectness	No serious imprecision	None	291	294	–	MD 0 higher (0.01 lower to 0.02 higher)	⊕⊕⊕○	Important
											Moderate	
**Resting heart rate (better indicated by lower values)**												
10	Randomized trials	Serious^a^	Serious^d^	No serious indirectness	No serious imprecision	None	243	251	–	MD 1.21 higher (0.28 lower to 2.71 higher)	⊕⊕	Important
											Low	
**Peak heart rate (better indicated by lower values)**												
12	Randomized trials	Serious^a^	Serious^e^	No serious indirectness	No serious imprecision	None	320	326	–	MD 6.86 higher (4.49 to 9.24 higher)	⊕⊕○○	Important
											Low	
**Oxygen pulse (better indicated by lower values)**												
4	Randomized trials	Serious^a^	Serious^f^	No serious indirectness	No serious imprecision	None	145	153	–	MD 0.97 higher (0.34 to 1.6 higher)	⊕⊕	Important
											Low	

a The included studies were biased in terms of allocation concealment and blinding.

b The results of the included studies were highly consistent.

c The results of the two studies were inconsistent.

d The results of the two studies were inconsistent.

e The results of the one studies were inconsistent.

f The results of the one studies were inconsistent.

The ⊕ symbol indicates the level of evidence quality. ⊕⊕⊕⊕ indicates the high level of evidence quality. ⊕⊕⊕ indicates the moderate level of evidence quality. ⊕⊕ indicates the low level of evidence quality. ⊕ indicates the very low level of evidence quality.

## Discussion

### Summary of the evidence

In this study, we selected 19 RCTs with a total of 1,036 patients. Our results show that compared with moderate-intensity exercise training, high-intensity exercise training has better improvement effects on Peak VO_2_, LVEF, ED, PHR and O_2_ pulse in patients with CAD. However, there are no significant differences in the effects of AT, RER and RHR. Furthermore, our subgroup analysis showed that there is no statistical difference in the influence of intervention time on peak VO_2_, RER and PHR. The effect of exercise intensity on ED and RHR was influenced by the intervention time. For the ED outcome, high-intensity exercise was superior to moderate-intensity exercise only when the intervention time was ≥12 weeks. For the outcome of RHR, when the intervention time was <12 weeks, high-intensity exercise has better improvement effects than moderate-intensity exercise. But when the intervention time was ≥12 weeks, the difference was not statistically significant.

### Results in relation to other studies

There were some relevant meta-analysis articles published lately, such as the recently published one by Gomes-Neto (44). However, the retrieval deadline of the study mentioned above was in November 2016. Only 12 literatures were selected in that study and the efficacy indicators of cardiopulmonary function were very limited (only peak VO_2_). On the contrary, our study screened all qualified literature from the establishment of the relevant databases to April 2022, and 19 RCTs were selected. We also analyzed more indicators related to cardiopulmonary function (peak VO_2_, AT, LVEF, ED, RER, RHR, PHR, and O_2_ pulse). Hence, our study provided more robust and comprehensive evidence for evaluating the effect of moderate and high exercise intensity on cardiopulmonary function.

### Potential mechanism

The underlying mechanism of high-intensity exercise improving peak VO_2_ in patients with CAD may be related to the fact that high-intensity exercise can stimulate muscle vascularization, improve blood circulation, and enhance blood oxygen-carrying capacity (45); In addition, studies (46) have shown that higher intensity exercise can stimulate the pumping capacity of the heart to a greater extent, increase blood flow, increase endothelial shear stress, activate endothelial nitric oxide synthase, and increase antioxidant status. Thus, nitric oxide synthesis is improved and its bioavailability is increased, which consequently improves the vascular endothelial function (47–49). High-intensity exercise can also increase oxisome proliferator-activated receptor γ coactivator 1α (a regulator of mitochondrial biogenesis) to improve mitochondrial function and enhance rapid adaptation and metabolic capacity in skeletal muscles (50). Therefore, high-intensity exercise is more effective in increasing Peak VO_2_ than moderate-intensity exercise training. LVEF is a major indicator of the pumping capacity of the heart. An elevated LVEF level indicates improved cardiac function. The mechanism of which exercise intensity improves LVEF is not clear, but it may be related to the ability of higher intensity exercise reduces left ventricular end-diastolic volume (EDV) and left ventricular end-systolic volume (ESV), improves ventricular remodeling and myocardial contractility. The PHR improvement in the high-intensity exercise group is better than that in the moderate-intensity exercise group, which can be attribute to the fact that higher exercise intensity can increase stroke volume, enhance myocardial contractility, increase the ejection fraction during extreme exercise, and improve exercise tolerance (51). ED is the duration of exercise from the beginning to the end of the evaluation in the Cardiopulmonary Exercise Test (CPET). The overall analysis showed that high-intensity exercise prolongs ED better than moderate-intensity exercise. However, our subgroup analysis results shows that when ≥12 weeks, the duration of high-intensity continuous exercise is longer than that of moderate-intensity. This implies that high-intensity exercise results in a better improvement in the exercise endurance of patients over time. Since PHR is affected by multiple factors such as age, gender, body size, muscle volume, daily activity level and exercise type (52), their specific mechanisms need to be further explored through more detailed and high-quality clinical trials. High-intensity exercise training is beneficial to anaerobic glycolysis and increases the lactic acid content in the blood. Lactate is converted back to glucose in the liver, thus AT is related to the gluconeogenic capacity of the liver. An experimental study in animal has shown that high-intensity exercise training can enhance the hepatic gluconeogenesis of lactate and increase the lactate threshold (53). However, this study shows that there is no significant difference in the effect of exercise intensity on AT, which may be due to the difference in lactic acid metabolism of human bodies. In addition, the concept of AT is still controversial, and the calculation methods, equipment used and detection personnel under various concepts will also have a significant impact on the results (54). RER expresses the relationship between carbon dioxide produced (CO_2_) and oxygen consumed (O_2_) which can be used to determine the rate of lipid oxidation (55). Studies showed that a reduction in the levels of RER levels after high-intensity exercise compared to moderate-intensity exercise (56, 57). However, our study did not find differences in the effect of exercise intensity on RER, which may be due to the lack of consideration for the effect of related gene expression on RER. Furthermore, some studies found that differences in exercise performance and muscle metabolic activity are associated with ACTN3 gene polymorphisms (58–60). RER decreases in subjects with only X allele after high-intensity exercise training, while there is no significant change in RER in RR homozygous subjects (61). High-intensity exercise has an advantage over moderate-intensity exercise in reducing RHR (62). And our study found that when the intervention time is <12 weeks, the RHR of high-intensity is slower than that of moderate-intensity, but there is no difference in intensity between the two exercises when it is ≥12 weeks. This may be attribute to the fact that high-intensity exercise is better at slowing PHR for a short period of time, but when patients keep exercising for longer time, the advantage of high-intensity exercise disappears.

## Limitations

Due to time and funding constraints, most of the current studies only observed the impact of exercise training on cardiopulmonary function indicators in patients. But there are few reports regarding long-term follow-up and clinical endpoint events as the salient endpoint indicators, which offers us a direction worthy of in-depth research in the future; The 19 studies adopted different exercise programs, and there are differences in race, gender and age among the subjects. In addition, exercise time (morning/ afternoon/ evening) and the total calorie consumption during exercise was not uniform, which may be responsible for the bias in our study. And there were large differences in the quality of the literature and the sample size of each study.

## Future directions

Due to the limitation of the sample size of the included studies, for patients with severe disease or patients with multi-vessel coronary artery disease, whether it is possible to continue to recommend high-intensity exercise programs is an interesting direction for future research. With the extension of exercise time, there are special changes in cardiopulmonary function indicators, which also provides an interesting direction for the design of future research duration. Given the limitations mentioned above, further high-quality studies are still needed to provide more reliable and higher-level evidence-based evidence on this subject matter in the future.

## Conclusion

Compared with moderate-intensity exercise training, high-intensity exercise training is more effective in improving peak VO_2_, LVEF, ED, PHR and, O_2_ pulse in patients with CAD. Nonetheless, there is no statistical difference in the effects of the two exercise intensities on AT, RER, and RHR. Among them, high-intensity exercise did not show an advantage in prolonging ED until intervention time reached 12 weeks. Also, high-intensity exercise is better at slowing RHR within 12 weeks, but this advantage disappeared with increased exercise duration.

## Data availability statement

The original contributions presented in the study are included in the article/supplementary material, further inquiries can be directed to the corresponding author.

## Author contributions

LZ, DP, and YG designed the study and assessed the risk of bias. LZ and DP analyzed the data and wrote the first and revised version of the manuscript. RW and YW screened and extracted the data. LZ, DP, and MX modified the final manuscript. All authors read and approved the final manuscript, contributed to the conceptualization of the research questions, interpretation of the results, and article writing.

## Funding

This work was supported by the National Key Programme for Research and Development from the Ministry of Science and Technology, China (No. 2019YFC0840608), National Natural Science Foundation of China (No. 81973686), and Major research project of scientific and technological innovation project of Chinese Academy of Chinese Medicine Sciences (No. CI2021A00913).

## Conflict of interest

The authors declare that the research was conducted in the absence of any commercial or financial relationships that could be construed as a potential conflict of interest.

## Publisher's note

All claims expressed in this article are solely those of the authors and do not necessarily represent those of their affiliated organizations, or those of the publisher, the editors and the reviewers. Any product that may be evaluated in this article, or claim that may be made by its manufacturer, is not guaranteed or endorsed by the publisher.
